# High-Degree Atrioventricular Block and Torsades De Pointes in Severe Aortic Stenosis Treated With Transcatheter Aortic Valve Replacement

**DOI:** 10.7759/cureus.63315

**Published:** 2024-06-27

**Authors:** Victor H Molina-Lopez, Benjamin A Gonzalez Burgos, Porfirio E Diaz-Rodriguez, Antonio L Orraca-Gotay, Luis Rodriguez-Ospina, Ismael Ortiz Cartagena

**Affiliations:** 1 Cardiovascular Medicine, Veterans Affairs Caribbean Healthcare System, San Juan, PRI; 2 Internal Medicine, Veterans Affairs Caribbean Healthcare System, San Juan, PRI; 3 Cardiovascular Medicine, Pavia Santurce Hospital, San Juan, PRI; 4 Interventional Cardiology, Veterans Affairs Caribbean Healthcare System, San Juan, PRI; 5 Interventional Cardiology, Pavia Santurce Hospital, San Juan, PRI

**Keywords:** paroxysmal complete heart block, critical aortic stenosis, recurrent ventricular tachycardia, polymorphic ventricular tachycrdia, torsades de pointes (tdp)

## Abstract

Severe aortic stenosis (AS) significantly elevates cardiovascular risk, predisposing patients to high-degree atrioventricular (AV) block and life-threatening tachyarrhythmias, including torsades de pointes (TdP). This case report presents a patient with severe AS who developed high-degree AV block and, subsequently, TdP, highlighting the interplay between bradycardia and mechanisms that trigger ventricular tachycardias. The case underscores the importance of identifying and managing these risk factors to improve patient outcomes.

## Introduction

Severe aortic stenosis (AS) is a critical condition characterized by the narrowing of the aortic valve, leading to significant hemodynamic consequences and increased cardiovascular risk. Among the various complications, patients with severe AS are predisposed to high-degree atrioventricular (AV) block and life-threatening tachyarrhythmias, including torsades de pointes (TdP) [1]. We present a patient with severe AS who developed a high-degree AV block and subsequently recurrent episodes of TdP. The case highlights the interplay between bradycardia and the mechanisms that trigger ventricular tachycardias in severe AS.

## Case presentation

**Figure 1:**
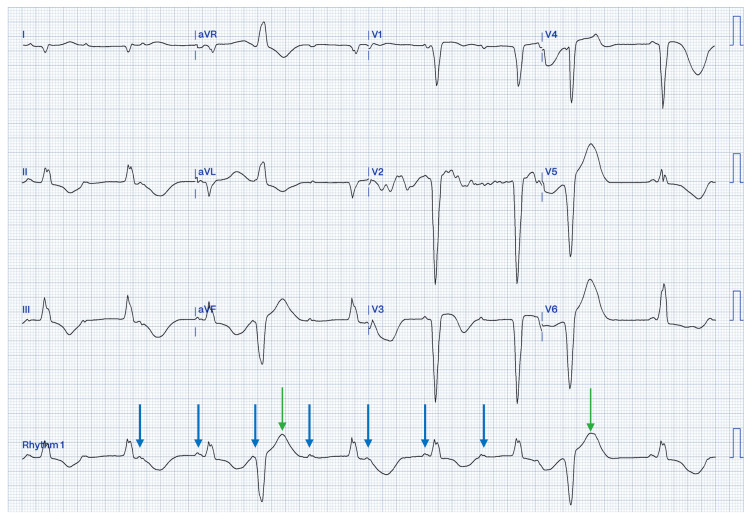
Initial electrocardiogram A 12-lead electrocardiogram demonstrating complete atrioventricular dissociation with a sinus rhythm at 60 beats per minute and junctional escape rhythm with a ventricular rate of 45 beats per minute (blue arrows). Junctional rhythm was remarkable for a nonspecific interventricular conduction delay. The green arrows demonstrate frequent ventricular ectopy very close to the prior T wave.

A 71-year-old male with no known past medical history presented to the emergency department (ED) because of multiple episodes of near syncope. The patient reported a progressive onset of generalized weakness and fatigue over the past several months. Additionally, he experienced six months of progressively worsening exertional dyspnea and angina with minimal activity. An initial evaluation in the ED revealed a complete AV block with frequent ventricular ectopy (Figure 1).

Upon initial assessment, vital signs revealed complete AV dissociation with a sinus rhythm of 60 beats per minute and a ventricular rate of 45 beats per minute, along with frequent ectopic ventricular beats. Blood pressure was 100/60 mmHg, and the respiratory rate was 18 breaths per minute. The patient was chest pain-free and reported no respiratory difficulties. There was no fever noted. The patient lacked skin lesions, a history of thick bites, and no history of camping, hiking, outdoor activities, or travel to an endemic area for Lyme disease. There was no physical exam finding about musculoskeletal or neurologic manifestations. Auscultation revealed a harsh, crescendo-decrescendo systolic ejection murmur characteristic of severe AS. This murmur was best heard at the right upper sternal border and radiated to the carotids. Notably, the second heart sound (S2) was inaudible. Additionally, the Galliverdin phenomenon was present, manifesting as a high-pitched, musical murmur heard at the apex.

He used no chronic medications or dietary supplements. Initial laboratory tests were within normal limits, and no reversible causes for the complete heart block were identified. Electrolytes were within normal range, thyroid function was normal, and serial high-sensitivity troponins were negative. B-type natriuretic peptide was 2,590 pg/mL. An admission chest radiography revealed aortic calcifications and mild cardiomegaly but no radiographic signs of pulmonary congestion. The transthoracic echocardiogram (TTE) demonstrated concentric left ventricular hypertrophy (LVH) with an ejection fraction of 60%, grade II diastolic dysfunction, and no regional wall motion abnormalities. Severe AS was identified, with an aortic valve area (AVA) of 0.5 cm², a maximum velocity (Vmax) across the valve of 5.10 m/s, and a mean pressure gradient of 60 mmHg. The aortic valve was tri-leaflet and had sclerosis and calcification. The rhythm during the study was of intermittent complete AV block with frequent ventricular ectopic beats.

**Figure 2:**
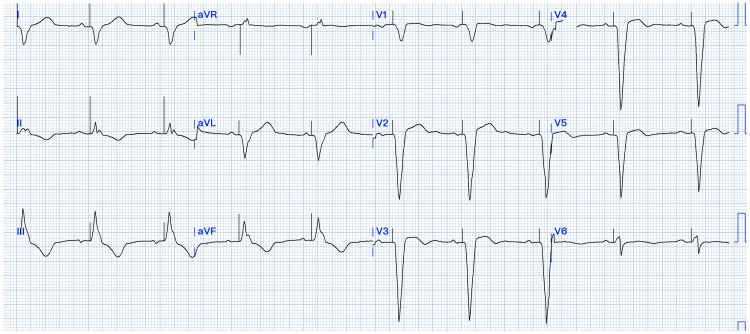
Electrocardiogram after leadless pacemaker implant A 12-lead electrocardiogram with sinus rhythm and ventricular paced complexes demonstrating adequate atrial sensing and ventricular pacing. The corrected QT interval while on paced rhythm was 500 ms.

A Micra AV transcatheter pacing system (Medtronic, Minneapolis, MN) leadless pacemaker (LPM) was successfully implanted per standard implant technique on the same day of admission. The procedure aimed for a high right ventricular septal placement of the device, which was achieved on the first attempt with initial R waves measuring 13.8 mV and an impedance of 970 ohms, and capture thresholds of 0.38 volts at 0.24 ms. Post-implant parameters were appropriate, and there was no evidence of pacing-induced ventricular arrhythmias on telemetry tracings. Post-implant electrocardiogram demonstrated adequate capture (Figure 2). The ventricular pacing percentage was 51.6%, as the complete AV block was intermittent.

**Figure 3:**
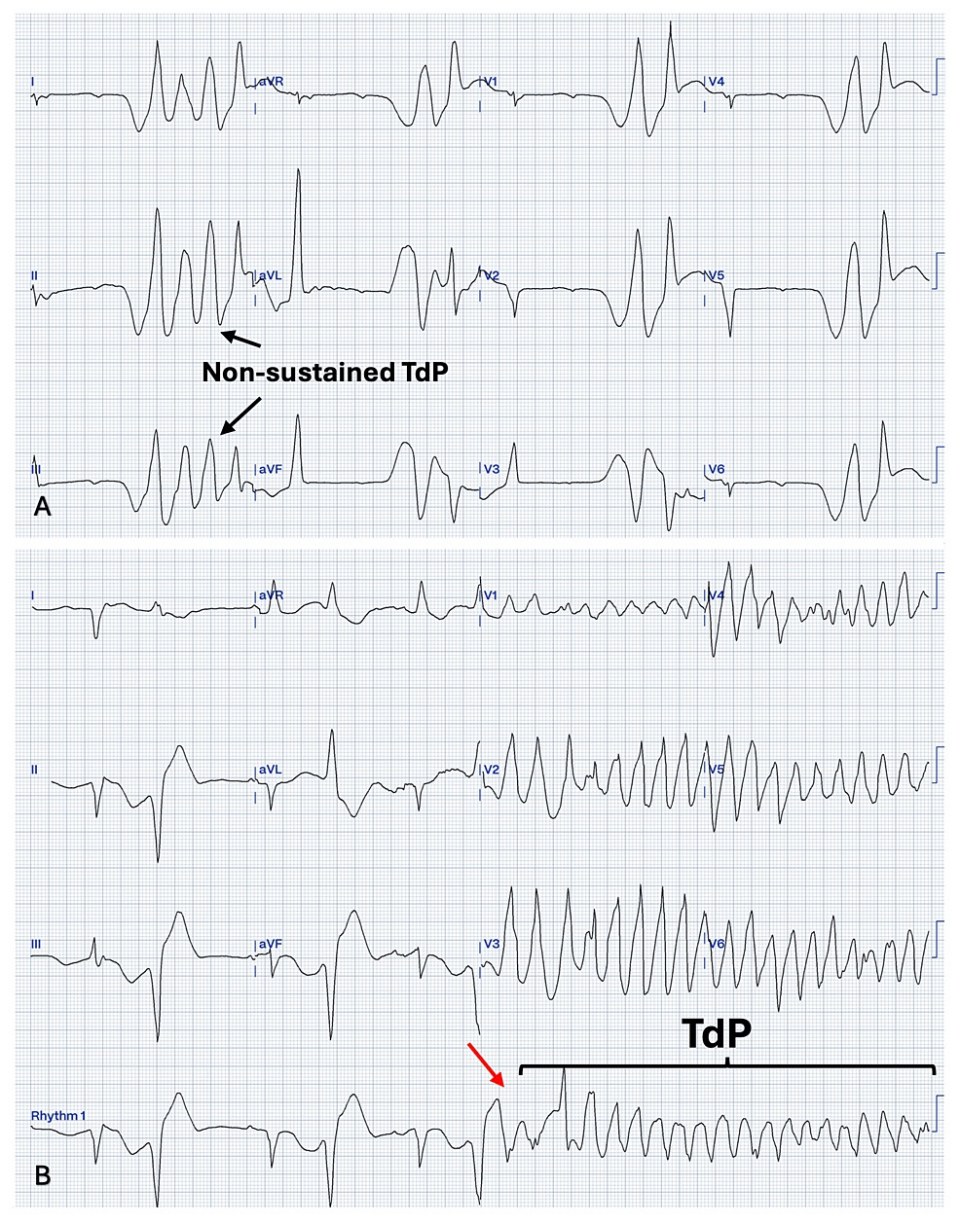
Electrocardiogram with episodes of ventricular arrhythmias and episodes of TdP (A) Episodes of nonsustained torsade the points (TdP). (B) Sinus rhythm with ventricular bigeminism with a fixed coupling interval. The frequent ventricular ectopic beats are depolarizing in the preceding T wave, causing R over T phenomena (red arrow) which starts a run of TdP.

Over the next two days, the patient remained admitted while undergoing evaluation and preparation for aortic valve replacement. Frequent ectopic ventricular beats and short rounds of polymorphic ventricular beats were present even before the LPM implant (Figure 3A). During admission, the patient experienced multiple episodes of polymorphic ventricular tachycardia suggestive of TdP (Figure 2B), necessitating unsynchronized cardioversion on multiple occasions. There was no major electrolyte derangement, and the patient was not on QT-prolonging medications.

Ventricular tachycardia was managed acutely with intravenous (IV) magnesium and lidocaine. He required endotracheal intubation, sedation, and neuromuscular paralysis with cisatracurium because of frequent episodes of TdP requiring cardioversion and recurrent cardiac arrests.

**Figure 4:**
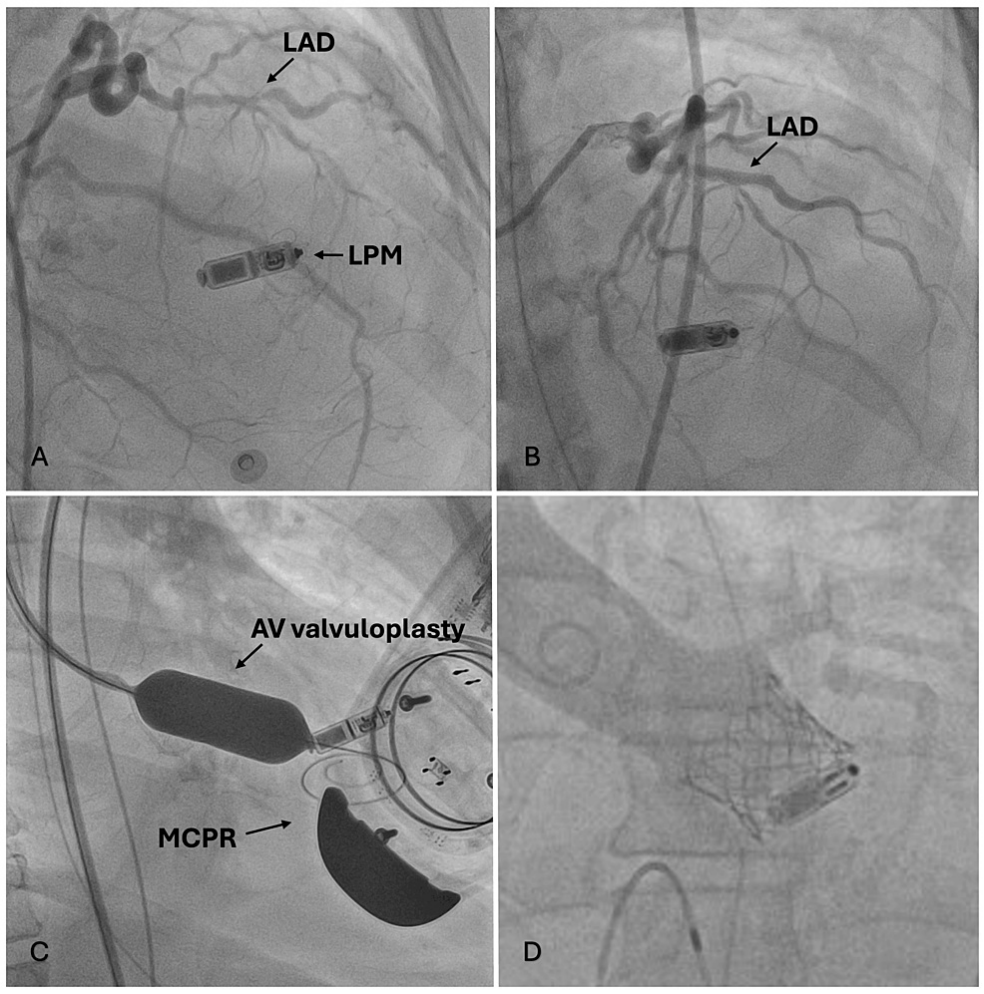
Coronary angiography during PCI with ad hoc AV valvuloplasty while undergoing MCPR for cardiac arrest (A) Coronary angiography revealing a mid-LAD obstructive lesion and (B) after PCI with a drug-eluting stent. Because of recurrent TdP, the patient went into cardiac arrest during coronary angiography, and (C) an emergent ad hoc AV balloon valvuloplasty was performed. (D) Salvage TAVR was successfully performed two days later with a balloon-expandable transcatheter aortic valve. LPM: leadless pacemaker; LAD: left anterior descending coronary artery; AV: aortic valve; MCPR: mechanical cardiopulmonary resuscitation device; PCI: percutaneous coronary intervention; TIMI: thrombolysis in myocardial infarction grade; TAVR: transaortic valve replacement

Emergent coronary angiography revealed single-vessel coronary artery disease with a focal 75% lesion in the mid-left anterior descending (mid-LAD) artery (Figure 4A). The mid-LAD lesion was treated with the placement of a drug-eluting stent (DES) (Figure 4B). Concurrently, because of the patient's severe AS, an ad hoc bridge balloon valvuloplasty was performed to address the severe valve narrowing in the setting of recurrent cardiac arrest events (Figure 4C). This procedure successfully decreased the peak-to-peak valve gradient to 30 mmHg with a subsequent decrease in the frequency of TdP events. After completing the CT angiography (CTA) TAVR protocol, and extensive family discussions, it was agreed to proceed with transcatheter aortic valve replacement (TAVR) as a potential salvage therapy. The procedure involved the placement of a 26 mm Sapiens Ultra Resilia (Edwards Lifesciences, Irvine, CA) valve via a transfemoral approach at an implantation depth of 90:10, achieving a residual mean gradient of 1 mmHg without any paravalvular leaks or complications at the arterial access sites.

Following this successful TAVR procedure, the patient experienced a complete resolution of his ventricular tachycardia events, with no recurrence during admission. He was successfully weaned from sedation, paralysis, mechanical ventilation, and IV lidocaine with complete recovery of his cognition within two days after TAVR. This improvement came despite multiple advanced cardiac life support (ACLS) events because of recurrent cardiac arrests. He showed steady and encouraging signs of recovery, regaining strength, which facilitated the start of physical therapy and tailored rehabilitation strategies.

The patient was provided with a wearable cardioverter‐defibrillator (WCD) to protect him from sudden cardiac death (SCD) upon discharge home two weeks after admission with close clinical monitoring. Follow-up visits at one and three months failed to demonstrate a recurrence of the ventricular tachyarrhythmias after TAVR. Ultimately, the patient did not require an implantable cardioverter-defibrillator (ICD) after shared decision-making with the patient and electrophysiology team, marking a significant turnaround from a highly complex clinical scenario. The patient ultimately had a complete recovery without residual effects from his cardiac events.

## Discussion

A high-degree AV block is a serious, potentially life-threatening condition in patients with severe AS. These patients often develop AV block even before undergoing TAVR because of intrinsic conduction disturbances and sclerosis within the cardiac conduction system [1,2]. Degenerative causes are common and are linked to advanced age, chronic hypertension, and diabetes mellitus. Infectious causes, such as Lyme carditis and endocarditis, should be evaluated, as an AV block may be reversible with appropriate treatment. Additionally, ischemic causes, such as inferior wall ischemia or myocardial infarction, can result in reversible AV block. An AV block induced by vagotonic influences tends to be transient and generally does not require cardiac pacing. Intermittent complete AV block leading to syncope or presyncope is frequently observed in patients with existing heart disease or baseline bundle branch block (BBB), but it can also occur in those without underlying heart disease or apparent conduction abnormalities [3].

Baseline conduction disturbances, particularly BBBs, are strong predictors of high-degree AV block in patients with severe AS. Right BBB (RBBB) and left BBB (LBBB) are prevalent in this population and significantly increase the risk of developing AV block. Approximately 35-40% of patients with preexisting RBBB undergoing TAVR develop high-degree AV block, necessitating permanent pacemaker (PPM) implantation [4,5]. Other conduction abnormalities, such as first-degree AV block, left anterior hemiblock, and intraprocedural AV block, further elevate the risk, with incidences reaching up to 50% [6]. Electrocardiographic evidence of LVH has also been associated with increased occurrences of high-degree AV block and LBBB, requiring prolonged monitoring and potential PPM implantation [4].

Bradycardia and high-degree AV block are significant contributors to the development of TdP, a potentially fatal form of polymorphic ventricular tachycardia. Bradycardia prolongs the QT interval, fostering conditions conducive to TdP, which is exacerbated by ventricular premature contractions causing compensatory pauses. Early afterdepolarizations (EADs) during the repolarization phase can trigger ventricular arrhythmias. In acquired long QT syndrome (aLQTS), bradycardia or pauses lead to impaired IKr, resulting in prolonged and variable repolarization. Unstable repolarization, indicated by T/U wave variability on the ECG, increases the likelihood of arrhythmia. TdP is also more prevalent in patients with structural heart diseases such as heart failure, myocardial infarction, and LVH, as well as those with congenital long QT syndrome (cLQTS) [7-10].

EADs are particularly likely in the presence of drugs that inhibit IKr, which is essential for cardiac repolarization. Hypokalemia, often seen with bradycardia, enhances these drugs' blocking effects on IKr, further promoting EADs and increasing the risk of TdP [11,12]. Increased spatial dispersion of repolarization across different heart regions also contributes to TdP. Chronic AV block leads to electrical remodeling, which heightens this dispersion, creating a substrate for reentrant circuits and increasing the risk of TdP [11,12]. This arrhythmic complication illustrates the interplay between structural cardiac abnormalities and arrhythmogenic risks, with sudden cardiac death in severe AS patients often attributed to ventricular arrhythmias, including TdP and ventricular fibrillation [13,14].

Ischemia can also cause TdP by prolonging the QT interval through mechanisms such as electrolyte imbalances, altered ion channel function, increased dispersion of repolarization, EAD, and autonomic nervous system imbalance [10]. In severe AS, the heart's fixed cardiac output and inability to increase stroke volume effectively leads to elevated left ventricular pressure and subendocardial ischemia. This increased pressure, along with forceful ventricular contractions, can activate cardiac vagal afferent fibers, triggering the Bezold-Jarisch reflex (BJR). The BJR can cause paradoxical bradycardia, vasodilation, and hypotension, further destabilizing hemodynamics. These extreme conditions worsen myocardial perfusion and increase left ventricular strain, contributing to the worsening of overall cardiac ischemic burden in severe AS and predisposing to the development of TdP [15,16].

Management of TdP or significant QT prolongation involves addressing precipitating factors, such as discontinuing offending drugs and implementing cardiac monitoring. Managing ischemia, correcting electrolyte imbalances and hypoxia, and maintaining potassium levels in the high normal range, are all crucial interventions. Immediate treatment includes intravenous magnesium sulfate and electrical cardioversion for prolonged episodes. For recurrent TdP resistant to initial treatment, increasing the heart rate with isoproterenol (isoprenaline) or transvenous pacing can suppress the arrhythmia. Intravenous lidocaine or phenytoin, although rarely necessary, can also be used. Lidocaine, a type 1B antiarrhythmic agent, reduces action potential and refractory period duration, thereby shortening the QT interval. This medication is well-known for managing ventricular arrhythmias, especially in ischemia. Antiarrhythmic drugs that prolong ventricular repolarization should be avoided [10].

## Conclusions

Severe AS significantly elevates the risk of high-degree AV block and TdP through mechanisms involving QT interval prolongation, bradycardia, and conduction disturbances. The pathophysiological basis of these arrhythmias is rooted in the prolongation of the QT interval, the facilitation of EADs, and increased spatial dispersion of repolarization. These factors create a highly arrhythmogenic environment, particularly in the presence of QT-prolonging drugs. Effective patient management necessitates a comprehensive understanding and identification of these risk factors. Key strategies include meticulous perioperative management, vigilant monitoring of electrolyte levels and QT intervals, and judicious use of medications. Implementing these measures is essential for mitigating the risks and improving outcomes for patients with severe AS. Furthermore, we must recognize arrhythmias as potential presenting symptoms of AS, with valve replacement as a definitive treatment for these patients.
